# New insights and potential clinical implications of the odds ratio product

**DOI:** 10.3389/fneur.2023.1273623

**Published:** 2023-10-09

**Authors:** Magdy Younes

**Affiliations:** Department of Medicine, University of Manitoba, Winnipeg, MB, Canada

**Keywords:** sleep depth, delta power, in obstructive sleep apnea, insomnia, hypersomnia, validation, sleep architecture, precision medicine

## Abstract

The odds ratio product (ORP) is a continuous metric of sleep depth that ranges from 0 (very deep sleep) to 2. 5 (full wakefulness). Its advantage over the conventional method recommended by AASM is that it discloses different levels of stage wake (sleep propensity) and different sleep depths within the same sleep stage. As such, it can be used to identify differences in sleep depth between subjects, and in the same subjects under different circumstances, when differences are not discernible by conventional staging. It also identifies different sleep depths within stage rapid-eye-movement sleep, with possible implications to disorders during this stage. Epoch-by-epoch ORP can be displayed graphically across the night or as average values in conventional sleep stages. In addition, ORP can be reported as % of recording time in specific ORP ranges (e.g., deciles of the total ORP range) where it produces distinct distribution patterns (ORP-architecture) that have been associated with different clinical disorders and outcomes. These patterns offer unique research opportunities to identify different mechanisms and potential therapy for various sleep complaints and disorders. In this review I will discuss how ORP is measured, its validation, differences from delta power, and the various phenotypes, and their postulated mechanisms, identified by ORP architecture and the opportunities for research to advance management of sleep-disordered breathing, insomnia and idiopathic hypersomnia.

## Introduction

**Figure 1:**
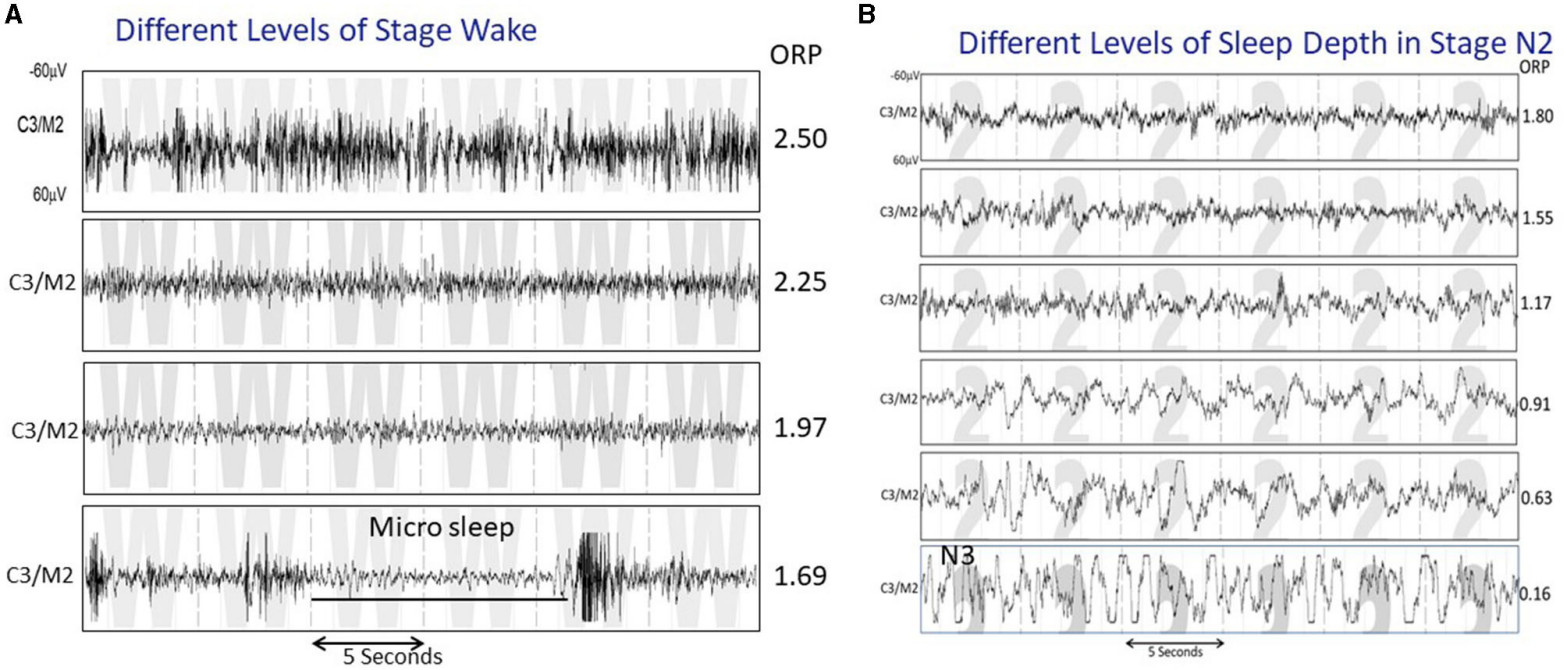
**(A)** Four 30-s strips of EEG tracings all staged as wake, illustrating various states between full wakefulness (top tracing) and near sleep. The ORP values reflect these differences. **(B)** Five 30-s strips staged as N2 but showing a variety of patterns that range from one that reflects very light sleep (top panel) to one that is very similar to stage N3 except that the total duration of delta waves is <20% of the epoch (From reference: Younes M, Azarbarzin A, Reid M, Mazzotti DR, Redline S. Characteristics and reproducibility of novel sleep EEG biomarkers and their variation with sleep apnea and insomnia in a large community-based cohort. *Sleep*. (2021) 44:145.).

In contrast to evaluating sleep state in discrete stages (wake, NREM1-NREM3), assigned every 30 s, the odds ratio product (ORP) measures wake/sleep state on a continuous scale from 0 (very deep sleep) to 2.5 (full wakefulness) and makes this assessment every 3 s (1). The continuous nature of the ORP scale makes it possible to distinguish different wake states in the transition from full wakefulness to light sleep (Figure 1A), and different levels of sleep depth within the same conventional sleep stage (Figure 1B). In addition, measurement over 3-s intervals makes it possible to measure brief dynamic changes in sleep depth that cannot be obtained from the conventional staging approach.

Since its original description in 2015 (1). ORP was used in numerous studies to determine normative values and reproducibility (2), relation to conventional staging (1, 3, 4), differences in ORP between central and frontal electroencephalogram (EEG) derivations (4), differences from delta power as measures of sleep depth (5), changes in obstructive sleep apnea (OSA) and Insomnia (6–8), sleep changes with continuous positive airway pressure (CPAP) (6, 9), changes in response to sleep restriction and deprivation (5, 10), maturational changes in sleep and association with pediatric psychiatric disorders (11, 12), association with CPAP adherence (13), association with sleepiness and quality of life (2, 7, 13, 14), underlying mechanism of poor sleep and its consequences in critically ill patients (15–17), response to traffic noise (18), association with traffic accidents (19), and dynamics of sleep recovery after arousal (8, 20).

Along with these studies, the reporting of ORP has evolved from simple description of its values in specific sleep stages or sleep disorders or as temporal changes across the night, to various patterns of ORP distribution within total recording time (7). This last development has changed ORP from being a simple descriptive tool of sleep depth to a way of understanding mechanisms of sleep complaints and disorders.

The above observations and developments have provided new insights into sleep physiology and pathology. However, virtually all published information was derived from retrospective studies. While the accumulated information is sufficient to formulate hypotheses about diagnoses and likely effective therapy of various sleep complaints, it is necessary to perform prospective studies to validate these hypotheses before ORP can be used clinically in patient management. In this review I will present the observations that form the bases for several proposed investigations, and what is needed to validate the retrospective observations. But first, some basic information about how ORP is measured, validation of ORP as a measure of sleep depth, and how ORP is reported, will be presented.

## How is ORP measured?

ORP can be measured from any central or frontal electrode (1, 4). This feature makes it possible to measure ORP from reduced monitoring devices attached to the forehead. Although only one derivation is needed, it is always better to monitor two similar electrodes, one on each side. This allows detection of differences in ORP between the two sides. Such differences make it possible to identify and discard artifacts and to detect true differences in sleep depth between the two sides, with potential clinical implications (2, 15, 19). In addition, one electrode can serve as a spare if the other fails.

The method of calculating ORP was described in detail elsewhere (1). Briefly, fast Fourier transform is applied to all EEG values within non-overlapping 3-s epochs. Total power in each of 4 frequency ranges, within the 0.33–35.0 Hz frequency range, is calculated. Power in each frequency range is assigned a rank from 0 to 9 based on its location within the range of powers (in the relevant frequency) observed in 56 clinical polysomnograms (PSGs) representing a wide range of clinical disorders. The four ranks are concatenated into one 4-digit number (Bin number) that describes the powers in the different frequencies from left to right *relative to each other*. Thus, 4,179 refers to a 3-s epoch in which power in the slowest range is in the 5^th^ decile of the range of powers observed in this frequency, while power in the next higher frequency is in the second decile, and power in the two highest frequencies are in their 8^th^ and 10^th^ (highest) deciles of their respective ranges [see Younes (3), for examples of EEG patterns with different bin numbers]. This approach is distinct from other spectral methods that rely on absolute power in selected frequencies, since absolute power is influenced by technical and biological factors unrelated to sleep depth [see, Normalized EEG power (2)]. The probability of patterns associated with each bin number occurring in epochs scored wake, or during arousals, is determined from a look-up table. This probability (0–100%) is divided by 40 (% wake epochs in development files) thereby converting the range to an ORP range of 0 to 2.5, where 0 refers to a pattern that never occurs during wake epochs or during arousals, while 2.5 refers to patterns that are never seen during sleep (1).

An important detail to note is that the slowest of the four frequency ranges used to calculate ORP (0.33–2.33 Hz) is different from the conventional delta range, which is wider (0.5–4.0, or 0.5–5.0 Hz) (21). For ORP, power in the fast delta range (2.6–4.0 Hz) is combined with power in the theta range (4.3–6.7 Hz) to provide the power in the second range used to calculate ORP. This has important implications to the EEG frequency that is most sensitive to sleep depth, as will be discussed in the section on ORP vs. Delta Power, below.

## Validation

**Figure 2:**
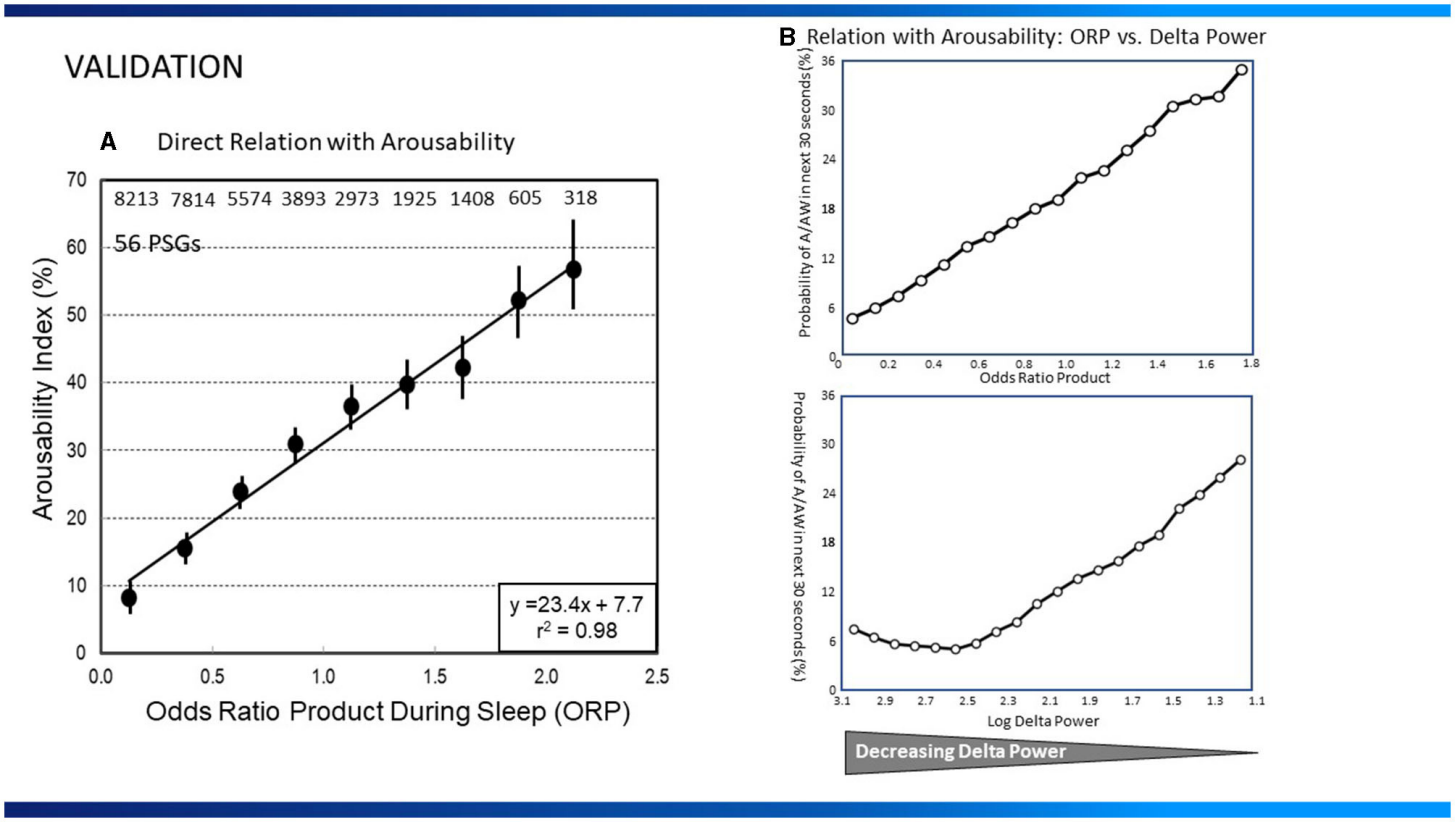
**(A)** Relation between average odds ratio product (ORP) in 30-s epochs during sleep and the probability of arousal or awakening in the next 30-s epoch (Arousability Index) in 56 polysomnograms (PSG) of patients with assorted sleep disorders. Numbers at top are number of 30-s epochs used at each ORP level (From reference: Younes M, Ostrowski M, Soiferman M, Younes H, Younes M, Raneri J, et al. Odds ratio product of sleep EEG as a continuous measure of sleep state. *Sleep*. (2015) 38:641–54.). Permission to be obtained if paper is accepted **(B)**, Top Panel: Relation between average ORP in 30-s epochs during sleep and the probability of arousal or awakening in the next 30-s epoch in 40 normal subjects. Bottom Panel: Relation between log delta power and arousal probability in the same sleep studies. Note that arousability decreases over a small range of delta power (From reference: Younes M, Schweitzer PK, Griffin KS, Balshaw R, Walsh JK. Comparing two measures of sleep depth/intensity. *Sleep*. (2020) 43:127.).

ORP correlates well with the visual appearance of the EEG (1, 7) (Figure 1), and decreases (deeper sleep) following sleep deprivation (10), and sleep restriction (5), while increasing as sleep progresses during the night (22). ORP increases transiently following application of brief noise stimuli whether or not they result in cortical arousal (18). However, the most compelling evidence is the finding that the correlation between ORP in a given 30-s epoch and probability of spontaneous arousal or awakening occurring in the next epoch is quite high (Figures 2A, B; *r*^2^ = 0.98) (1, 5).

## ORP vs. Delta power

Power in the delta frequency range (up to 4 or 5 Hz) is commonly used to evaluate sleep depth. However, other than its increase following sleep deprivation (23, 24), and decrease across the night (24), and during nocturnal sleep after daytime naps (25), there has been little information on the quantitative relation between delta power and sleep depth as defined by ease of arousing from sleep.

In a recent study (5), the relationships between delta power (0.33–5.67 Hz) or ORP and arousability, were compared in healthy young adults monitored overnight for 8 consecutive nights of which the first two served as baseline. Baseline results are shown in Figure 2B. The relationship between ORP in any given 30-s epoch and probability of arousal or awakening in the next 30-s epoch was, as described earlier (1), linear (*r*^2^ = 0.99; Figure 2B). The relationship for delta power was strikingly different; arousability decreased as delta power increased but only over a very limited delta power range (0.0–300 μV^2^), with no further decrease in arousability as delta power increased to 1,000 μV^2^ or more (Figure 2B). The inflection point, at 300 μV^2^, is generally the delta power at which the large delta waves (>75 μV^2^, 0.5–2.0 Hz) begin to appear and stage N3 is scored (5). Further increases in average delta power (in 30-s epochs) simply reflect increasing number and/or amplitude of delta waves. These observations have important *clinical implications*:

- Differences in delta power when delta waves are present do not reflect differences in sleep depth; rather they reflect density (number per minute) and amplitude of delta waves. Growing evidence points to a critical role of these delta wave characteristics in memory, cognition, sleep maintenance and mental health (26). Given that these characteristics can be easily measured in clinical sleep studies, reporting them, or simply reporting average delta power in stage N3, may provide important insights into mechanisms of various manifestations of clinical sleep disorders.
- Almost the entire change in sleep depth occurs before N3 is scored (Dotted vertical lines, **Figure 4**). Thus, absence of stage N3 need not signify absence of deep sleep.

## ORP and conventional sleep stages

**Figure 3:**
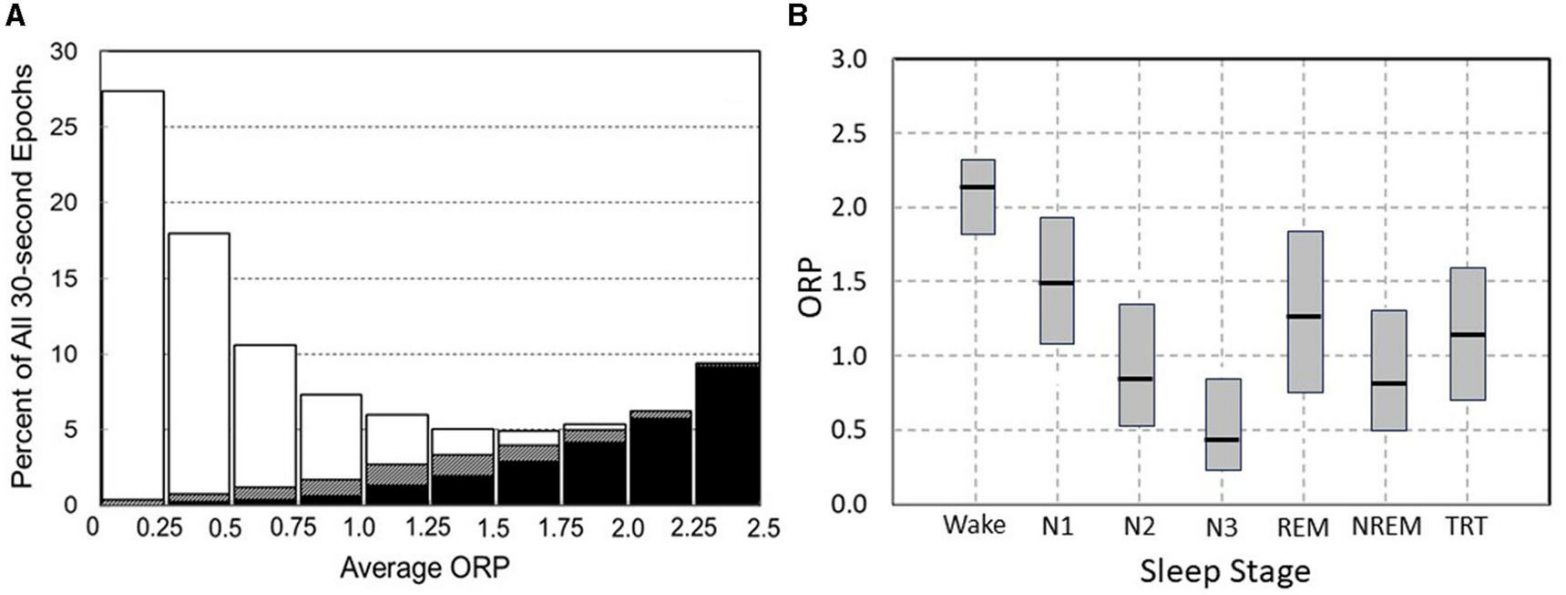
**(A)** Frequency distribution of 30-s epochs with different average odds ratio product (ORP). Within each bar white and black segments are epochs staged asleep and awake, respectively, by two expert technologists while hatched segments are epochs receiving a split awake/asleep decision (From reference: Younes M, Ostrowski M, Soiferman M, Younes H, Younes M, Raneri J, et al. Odds ratio product of sleep EEG as a continuous measure of sleep state. *Sleep*. (2015) 38:641–54.). **(B)** Range (median and 5 and 95 percentiles) ORP in different visually determined stages in 5,781 subjects of the Sleep Heart Health Study (SHHS) incorporation subjects with obstructive sleep apnea (OSA) (*n* = 2,504), insomnia (*n* = 419), insomnia + OSA (*n* = 403), and neither insomnia nor OSA (*n* = 2,455).

Figure 3A illustrates how 30-s epochs with different ORP levels are typically scored by expert scorers (Panel A), and Figure 3B the range of ORP in different visually scored sleep stages. When ORP is <0.25 two scorers agree that the patient is asleep almost all the time (white sections). When ORP is >2.25, they agree the patient is awake almost all the time (black zones). Percent of epochs scored wake by both scorers remains very low (<5%) until ORP of 1.00, but disagreement (gray zones) increases slightly as ORP approaches 1.00. Between ORP 1.00 and 1.75, disagreement between scorers occurs in a substantial fraction of epochs and for these three deciles collectively, the chances of disagreement, agreement on stage wake or sleep are almost random. Epochs in this range contain features of both stages to a sufficient extent that elicits disagreement between expert scorers. Accordingly, they are considered transitional. Between 1.75 and 2.25 epochs are most commonly scored wake by both scorers but in some epochs, sleep features (slowing, micro sleep) are sufficiently prominent to result in the epoch scored sleep by one or both scorers. Thus, ORP in this range reflects the extent to which visually appreciated sleep features exist in the epoch. Figure 3B shows results of ORP recorded in different stages in in the Sleep Heart Health Study (*n* = 5,804). There is a wide range of ORP in all stages but on averages ORP decreases progressively as stage progresses from wake to stage N3. The range of ORP in rapid eye movement sleep (REM) is very wide and on average higher than in stages N2 and N3. However, much overlap exists between stages.

## How is ORP reported and how its results might be interpreted?

While the methods of reporting are extensively described here, the interpretations suggested in this section are mostly based on retrospective studies or logical extension of basic sleep findings in the literature. Interpretations provided here are intended to stimulate discussion and to suggest ideas for prospective research and not as a guide to management.

**Figure 4:**
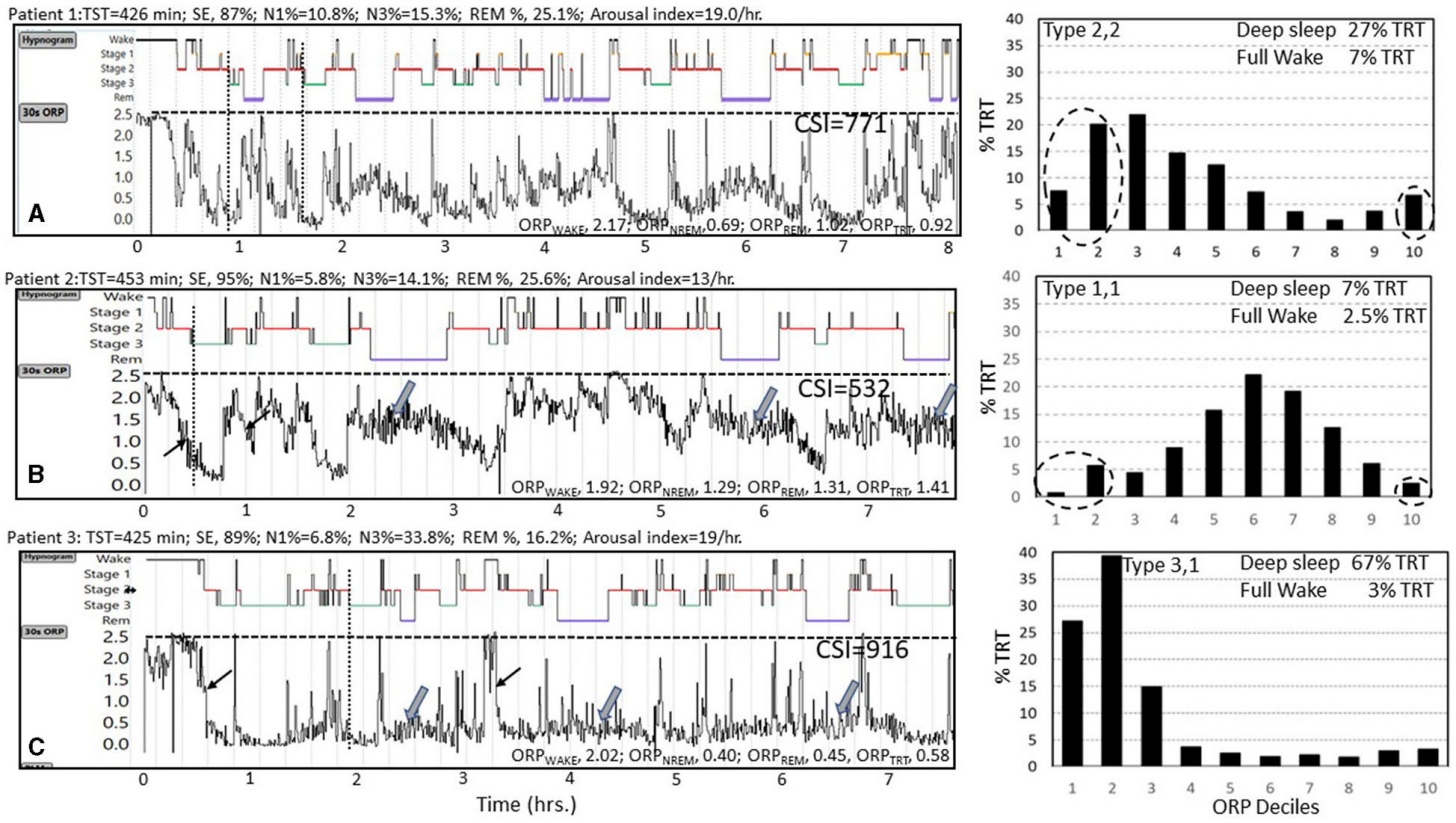
Data from three subjects **(A–C)** with normal conventional hypnograms illustrating substantial differences in their ORP metrics. Values above each panel are derived from the conventional sleep metrics showing that all values were within normal limits. TST, total sleep time; SE, sleep efficiency; N1%, N3%, and REM% are percent of TST in stages N1 and N3 of non-rapid eye movement sleep (NREM) and in rapid eye movement sleep (REM), respectively. Epoch by epoch odds ratio product (ORP) values are displayed as graphs below the hypnograms of the 3 subjects and as averages in different sleep stages and show substantial differences between the 3 subjects. ORP_WAKE_, ORP_NREM_, ORP_REM_, and ORP_TRT_ are the average values of ORP in all epochs staged as Wake, NREM sleep, REM sleep, and total recording time, respectively. Note that the rate at which ORP decreases from full wakefulness to deep sleep differs between subjects [thin arrows in subjects **(B, C)**], and that ORP during REM sleep varies among subjects [thick arrows in subjects **(B, C)**]. CSI, integral of the difference between 2.5 (full wakefulness) and instantaneous ORP (Graph values) across total recording time, representing total units of wake suppression during the study. Note the marked difference between the three subjects. Histograms to the right show %TRT spent within each ORP decile with sleep depth decreasing from decile 1 (very deep) to decile 7 (transitional sleep) to full wakefulness (decile 10). Note the marked difference in the ORP histograms of the three subjects with subject A having a normal distribution, subject B having most epochs in transitional and light sleep while in subject C the distribution is markedly shifted to the left. See text for interpretation of these differences (Un-published data).

ORP can be reported in several ways with each offering different insights into the patient's sleep. Figure 4 illustrates four of these approaches. The figure shows results of 3 subjects, one with no sleep symptoms (Panel 4A) and two with symptoms of insufficient/non-restorative sleep (Panels 4B and 4C). The conventional hypnogram and conventional architecture data (above each panel) in all three cases were within normal limits:

A) Graphical approach (30-s epoch-by-epoch ORP graph, Figure 4): This display provides a bird's-eye view of the changes in wake propensity and sleep depth across the night, offering detail that cannot be appreciated from numerical summaries. It also serves a learning objective in that it confirms some of the advantages of ORP. For example, in the illustrated Figure 4, one can see that ORP varies widely within stage wake and stage N2 (any panel), and that REM ORP may be higher (Figures 4A, B) or similar to ORP in NREM sleep in different patients (Wide arrows). It is also clear that ORP during stage N2 can be as low as in stage N3 (1, 5), such that absence of stage N3 does not mean lack of deep sleep. It can also be appreciated that the rate at which sleep deepens following an awakening is different among patients (compare rates of decline at light arrows in panels 4B and 4C). A curious observer may also make new connections between certain patterns and clinical presentations that may result in new research hypotheses.

B) ORP in Different Sleep Stages (Figure 4, values at the bottom of each panel): These values compliment conventional stages by showing differences between patients, or in the same patient before and after interventions, that cannot be disclosed by conventional stages. Figure 4, and the following discussion, illustrate how the use of ORP can identify sleep abnormalities when a patient is symptomatic, but the sleep study is normal by conventional metrics. Thus, notwithstanding the similar conventional architecture among the 3 subjects of Figure 4, ORP_WAKE_ was low (less alert) in subjects B and C than in subject A and ORP_NREM_ and ORP_REM_ were highest (lighter sleep) in subject B and very low (deep sleep) in subject C (Figure 4). The obvious next question is: what are normal values in the different sleep stages?

Table 1 shows average and range of ORP in different stages according to demographic and disease categories (2). ORP in any stage ranges widely among individuals within any category, even in subjects with no OSA or insomnia, and there is almost complete overlap in the ranges among different subcategories, so that there is no clear demarcation between values in health and disease. This is likely because sleep depth (ORP during sleep) and propensity (ORP during stage wake) are to a large extent influenced by sleep pressure (5, 7, 10). In turn, sleep pressure may be high or low in different subjects because of sleep pathology (e.g., excessive sleep need in idiopathic hypersomnia and hyperarousal in insomnia, respectively) or because of different demographics (2, 7), and sleep history (5, 7, 10) in people who are otherwise normal (Table 1). Accordingly, actual values in individual patients are not very helpful in determining, *per se*, whether they represent pathology or physiology.

**Table 1:** Odds ratio product in different stages per demographics and disease categories.

**Category**	**Number**	**ORP_WAKE_**	**ORP_NREM_**	**ORP_REM_**	**ORP_TRT_**
**All**	5,781	2.12 (1.85–2.35)	0.83 (0.49–1.22)	1.28 (0.74–1.89)	1.14 (0.73–1.60)
**Age (yrs.)**				
40–55	1,533	2.07 (1.74–2.30)^a^	0.79 (0.48–1.16)^a^	1.18 (0.72–1.75)^a^	1.04 (0.67–1.46)^a^
55–70	2,399	2.13 (1.83–2.34)^b^	0.83 (0.52–1.24)^b^	1.30 (0.78–1.86)^b^	1.13 (0.74–1.58)^b^
70–90	1,849	2.15 (1.84–2.36)^c^	0.87 (0.52–1.31)^c^	1.35 (0.79–1.92)^c^	1.22 (0.79–1.68)^c^
**Gender**				
F	3,027	2.14 (1.85–2.35)^a^	0.82 (0.50–1.25)^a^	1.28 (0.74–1.88)^a^	1.12 (0.71–1.60)^a^
M	2,754	2.09 (1.77–2.32)^b^	0.85 (0.53–1.25)^b^	1.29 (0.78–1.83)^a^	1.15 (0.75–1.59)^b^
**Race**				
White	4,889	2.13 (1.83–2.34)^a^	0.84 (0.51–1.25)^a^	1.28 ( 0.76–1.86)^a^	1.14 (0.73–1.60)^a^
Black	512	2.10 (1.77–2.33)^b^	0.88 (0.57–1.29)^b^	1.38 (0.88–1.93)^b^	1.20 (0.80–1.65)^b^
Other	380	2.05 (1.74–2.29)^c^	0.77 (0.49–1.13)^c^	1.18 (0.72–1.79)^c^	1.04 (0.67–1.47)^c^
**Disease category**				
No OSA or Insomnia	2,454	2.13 (1.83–2.35)^a^	0.81 (0.49–1.22)^a^	1.27 (0.74–1.89)	1.10 (0.70–1.56)^a^
Insomnia	419	2.16 (1.86–2.36)^b^	0.85 (0.49–1.29)^bc^	1.31 (0.78–1.92)	1.18 (0.73–1.65)^bd^
Comisa	403	2.13 (1.78–2.34)^ac^	0.88 (0.53–1.36)^b^	1.30 (0.77–1.83)	1.21 (0.78–1.66)^b^
Mild OSA	1,557	2.11 (1.80–2.33)^c^	0.83 (0.52–1.22)^acd^	1.28 (0.77–1.83)	1.13 (0.75–1.57)^c^
Mod. OSA	482	2.10 (1.80–2.33)^c^	0.85 (0.53–1.26)^bd^	1.28 (0.79–1.85)	1.15 (0.73–1.64)^cd^
Sev. OSA	465	2.07 (1.70–2.30)^d^	0.93 (0.58–1.35)^e^	1.3 (0.84–1.86)	1.21 (0.80–1.63)^b^

^*^, values are means (5–95 percentile).

a, b, c values within the same category that do not share the same superscript are significantly different from each other (*p* < 0.001).

ORP, odds ratio product; REM, rapid eye movement sleep; NREM, non-REM; TRT, total recording time. Values in this table were derived from Tables 2, 3 of reference Younes et al. (2).

What is important in interpreting ORP in sleep stages is to note where the value falls within its respective range in the community at large. A high ORP_NREM_ within its range (e.g., patient B, Figure 4; 1.29 in a range of 0.50–1.36, Table 1) indicates that NREM sleep is very light. Absence of clinical sleep symptoms (insomnia, excessive sleepiness, non-restorative sleep…etc.) would suggest a physiologic reason and may be ignored. On the other hand, presence of sleep symptoms (e.g., patient B, Figure 4) would point to: (a) A disorder that interferes with progression to deep sleep (e.g., OSA, periodic limb movement (PLM) disorder, other somatic or environmental arousal stimuli); (b) A low sleep pressure state associated with insomnia (hyperarousal) or related to lifestyle or use of stimulant drugs or drinks. These possibilities can be distinguished by other findings in the sleep study (e.g., OSA, PLMs, excessive wake time) or in the history (insomnia, lack of excessive somnolence, excessive napping, drugs, or stimulant drinks). Noting ORP in other sleep stages can be helpful in difficult cases. For example, high ORP_NREM_ associated with high ORP_WAKE_ suggests a low sleep pressure state, while an associated low ORP_WAKE_ points to a sleep disorder that interferes with progression to deep sleep (Patient B, Figure 4) (2, 8, 14).

By contrast, a low ORP_NREM_ (Patient C, Figure 4) could be normal, particularly in asymptomatic young adults (7). However, if associated with excessive somnolence or non-restorative sleep, it suggests a state of high sleep pressure due to insufficient sleep prior to the sleep study or excessive sleep need (certain types of idiopathic hypersomnia) (27). These can be distinguished from the sleep history.

Table 1 shows that on average ORP in all stages increases with age. ORP_WAKE_ is higher and ORP_NREM_ is lower in females, while the opposite is true in the black race. These differences are, however, small relative to the wide range in any category.

ORP_WAKE_ reflects the weighted average of ORP in all epochs scored wake. Thus, it is low when most wake epochs are in a drowsy wake state, indicating reduced vigilance (Figure 1A), and vice versa. Reflecting this, ORP_WAKE_ is higher in insomnia than in subjects with no insomnia while it decreases progressively with OSA severity (Table 1). By contrast, ORP_NREM_ is higher than controls (no OSA or insomnia) in the presence of both OSA and insomnia.

ORP_REM_ documents the variable background EEG in this stage, which visually ranges from a pattern indistinguishable from stage wake to one not that different from deep stage N2, without the spindles. Reflecting this range, ORP_REM_ is higher than ORP_NREM_ (0.74–1.89, Table 1) but the difference between ORP_NREM_ and ORP_REM_ varies widely from being minimal (Figure 4C) to being large (Figures 4A, B). The significance of these differences is not clear although the association of high ORP_REM_ with reduced REM time and increased REM fragmentation (28) may be relevant to abnormal dream states and mood disorders. Interestingly, ORP_REM_ is a strong trait (2) and, unlike other ORP measures, is not different between genders or disease phenotypes (Table 1).

In addition to the above uses of ORP in different stages, ORP_NREM_ was recently found to be a significant determinant of sleep improvement on CPAP (6) and adherence to CPAP (13), both outcomes are better when ORP_NREM_ is high before therapy.

### Opportunities for research

- The most significant advantage of ORP over conventional staging is its ability to identify differences in sleep depth within the same conventional sleep stage (Figure 1). An important clinical question is therefore whether clinical outcomes are improved when ORP in different stages is available to treating physicians investigating suspected sleep disorders. Specific questions may include: (A) Does ORP help identify abnormalities in patients with sleep complaints when conventional architecture is normal or inconclusive (e.g., using the approach described for interpreting differences in Figure 4)? (B) Does ORP help explain symptomatic improvement or deterioration following a given intervention (e.g., CPAP, or therapy for insomnia or depression …etc.) when conventional architecture did not change? For example, did ORP_NREM_ improve or deteriorate despite unchanged times in different sleep stages, or did ORP_WAKE_ increase or decrease, indicating change in sleep pressure, on therapy.
- Examining potential associations of ORP_REM_ with psychiatric disorders.
- Confirming the ability of ORP at baseline to predict sleep improvement on, (6) and adherence to, (13) CPAP.

C) Cumulative Sleep Index (CSI) (7): Given that ORP in full wakefulness is close to 2.5, the difference between 2.5 and ORP at any moment is a measure of “wake suppression” at that moment (Figure 4). CSI is the integral of these differences across total recording time (TRT) in minutes. Thus, a patient who remains fully awake throughout would have a CSI of 0 while a patient who was in very deep sleep throughout would have a CSI of 2.5^*^TRT. For a common TRT of 480 min, the maximum CSI is 1,200 min.ORPunits.

The advantage of CSI over total sleep time (TST) or sleep efficiency (SE) is that it takes into account different sleep depths during sleep and also includes reductions in ORP during stage wake (drowsy wake, Figure 1A). Thus, a minute with ORP of 1.8 during stage wake contributes 0.7 units to CSI when it does not contribute to TST or SE. Its advantage over ORP in total recording time (ORP_TRT_) is that it incorporates differences in TRT. In practice CSI is calculated from [(2.5-ORP_TRT_) ^*^ TRT] (7). When CSI is measured from sleep studies with unrestricted time in bed, it provides the total “units of sleep” needed by the subject to sleep enough (i.e., individual sleep need), particularly if the value is reproducible over several consecutive nights.

**Figure 5:**
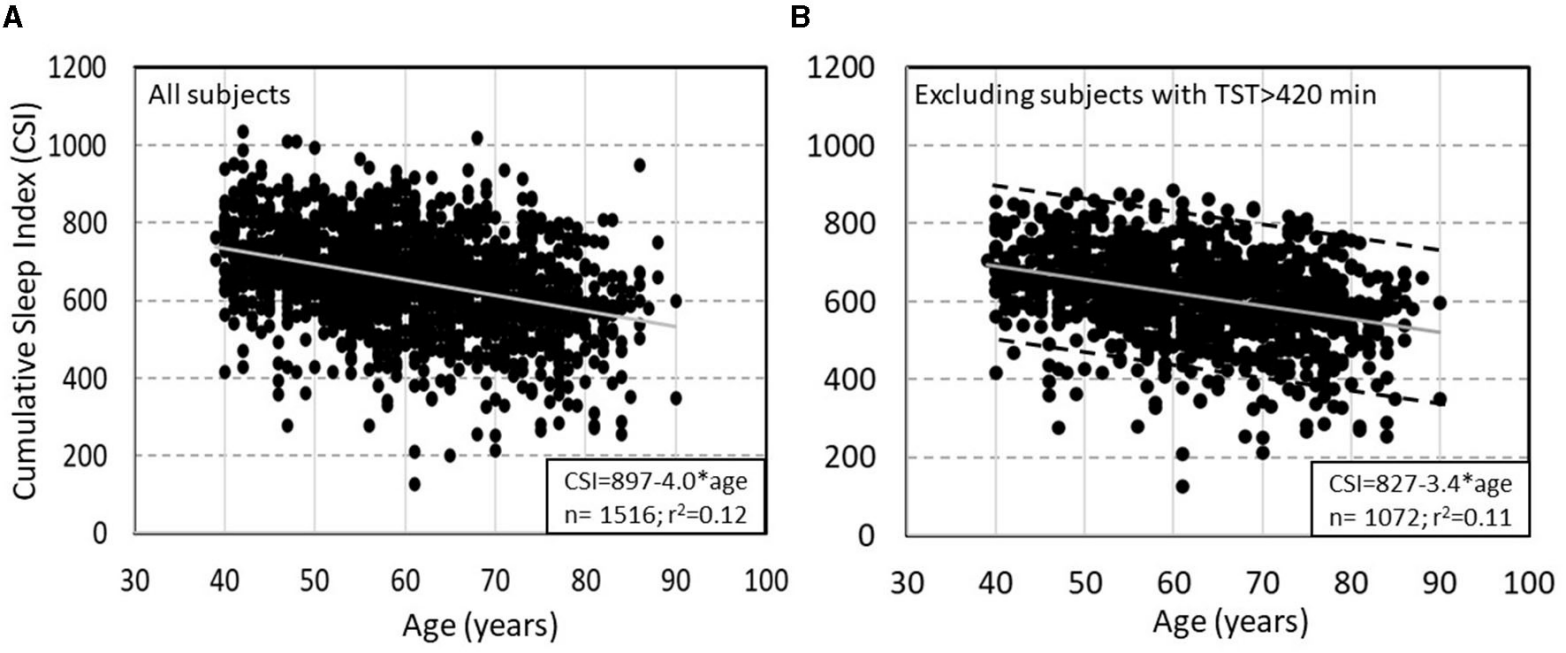
**(A)** Relation between Cumulative Sleep Index (CSI) and age in subjects of the Sleep Heart Health Study (SHHS) with total recoding time >7 h and no obstructive sleep apnea or insomnia. **(B)** Same relation after excluding subjects with >7 h of total sleep time. Dashed lines in panel B are ±2 SD from the main regression line (un-published data).

Normal values have not yet been properly established. However, the Sleep Heart Health Study (SHHS) provides preliminary data on this variable in that subjects were not instructed to wake up at any specific time. In SHHS subjects with no OSA or insomnia and with TRT >7 h (to avoid studies ending because of technical problems) TRT, TST, and SE ranged up to 541 min (≈9 h), 519 min (8.7 h), and 98.4%, respectively. In this cohort CSI averaged 651 ± 129 and was reported to decline with age (due primarily to declining ORP_TRT_) and to be only marginally higher in women, and not affected by BMI (7). The most interesting finding here was that at any age the range of CSI varied widely with a SD of 121 units, representing a 90% confidence interval of 484 units (Figure 5A). After excluding 444 subjects with TST>7 h, who likely had excessive sleep pressure during the single study, the relation with age was essentially unchanged with the exception that the standard deviation (SD) decreased from 121 to 108 (Figure 5B). This wide variation of “sleep need” is particularly noteworthy as it is not consistent with the fairly narrow range of currently recommended sleep time (7–8 h).

**Figure 6:**
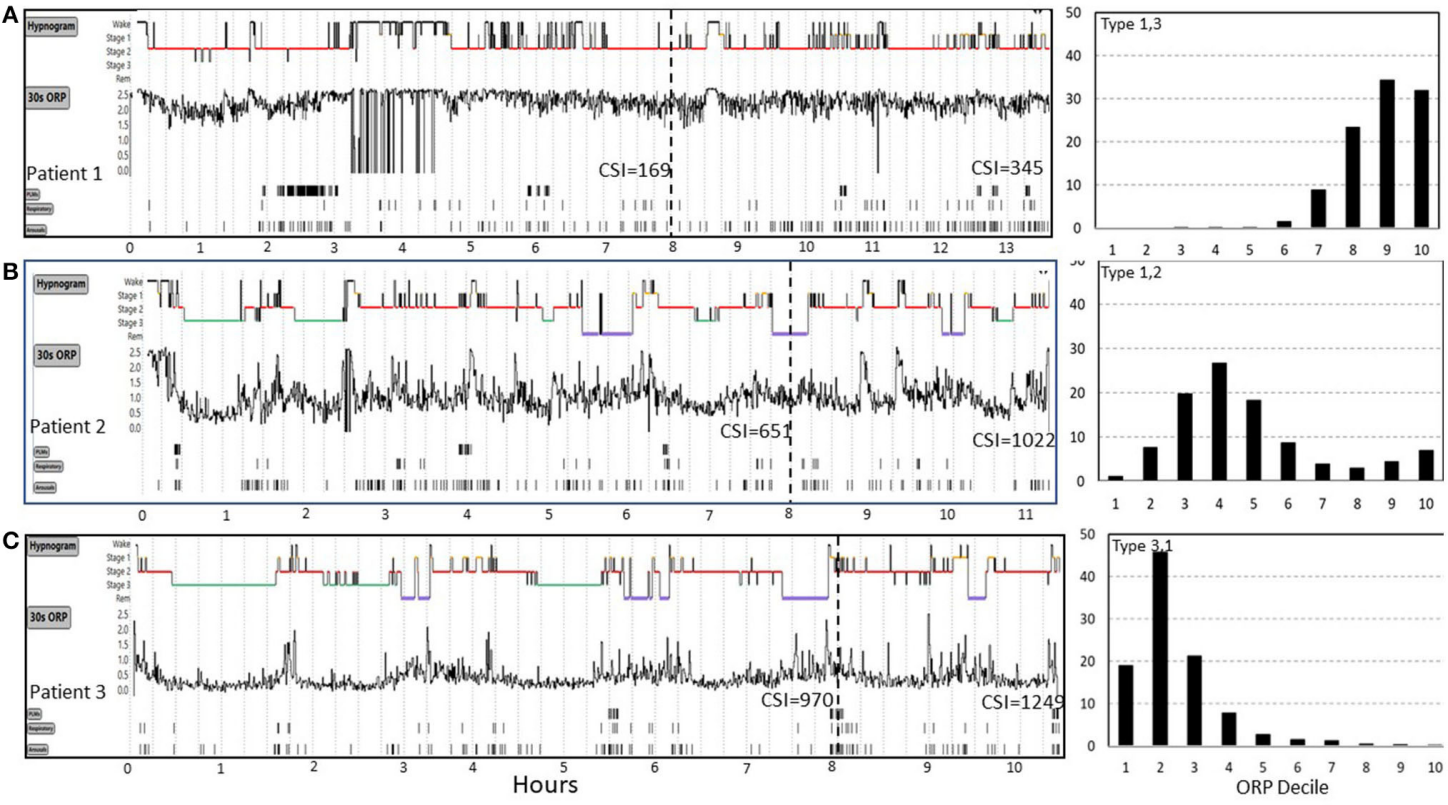
Range of odds ratio product (ORP) results in three patients (patients 1, 2, 3) with idiopathic hypersomnia represented in **(A–C)**. Cumulative sleep index values (CSI) values are shown at 8 hours and at the end of the study. Patient 1 had very high ORP (light/transitional sleep) throughout the 13 h study. His CSI was only 169 at 8 h and was still 345 after 13 h, both values are well below average sleep need (cf. Figure 5). CSI in patient 2 was normal at 8 h but increased well above mean ±2 SD (Figure 5B) after 11 h of sleep, indicating high sleep need. In patient 3 CSI was already well above average (970) at 8 h but increased further to 1,249 at 10.5 h, indicating extremely high sleep need. ORP histograms are shown to the right and illustrate marked differences despite the same clinical diagnosis. PLM, periodic limb movements. See text for potential implications of these different patterns on management (From an unpublished study by Dr. Robert Thomas, with permission from Dr. Thomas).

CSI was recently used to investigate mechanisms of idiopathic hypersomnia (IH) (27). Figure 6 shows results from 3 patients from this study who underwent sleep studies with unrestricted duration in Dr. Robert Thomas' laboratory at Harvard University and represent the extreme range of the results. All three patients slept >10 h (Figure 6), had no OSA (apnea hypopnea index (AHI) 2.4, 0.9, and 0.5 h^−1^), and only one patient had brief periods of PLMs (Figure 6A) (PLM index 26, 3, and 3 h^−1^), thereby consistent with IH.

Arousal index was normal in all three (12, 22, and 21 h^−1^) and although total sleep time (TST: 319, 395, and 474 min, in patients 1–3) and sleep efficiency (SE:70, 84, and 95%) were different among the three patients in the first 8 h, the differences were not informative regarding the reason for excessive sleepiness. The ORP tracings (Figure 6) clearly show that sleep was very light in patient 1, deeper in patient 2, and even deeper in patient 3 (ORP_TRT_: 2.13, 1.12, and 0.48, respectively), thereby indicating that insufficient sleep in a typical time in bed (8 h) may be contributing to sleepiness in patient 1 but not likely in patients 2 or 3, unless sleep need is high (i.e., idiopathic hypersomnia).

CSI provides additional information to what can be gleaned from ORP values in that it is a quantitative index of how much sleep the patient obtained in the usual 8-h study vs. what he/she gets with unrestricted sleep. Thus, Patient 1 managed only 169 units of sleep at 8 h, well below the 90% CI observed in community dwellers (Figure 5). At the end of the unrestricted study CSI was 345, well below average sleep need (e.g., Figure 5). Given these findings, it may be reasonable to conclude that, rather than having excessive sleep need, this patient has decreased sleep need (e.g., hyperarousal) as evidenced by the low CSI after ad lib sleep, while this very modest sleep need cannot be delivered in 8 h due to the very poor sleep quality. Thus, pending validation studies, investigation and treatment of poor sleep to lower ORP might be the appropriate management strategy in this patient.

Patient 2 (Figure 6) had an average amount of sleep in the first 8 h (CSI 651) but he clearly needed more (1,022 at the end of the study). At his average ORP rate (ORP_TRT_ = 1.12), he needed an extra 4 h. However, his ORP during the first 8 h had still some room to improve (decrease). If ORP could be decreased to the same level as patient 3 (ORP_TRT_ = 0.48) patient 2 can potentially achieve his needs [1,022] within a normal time in bed (Patient 3 achieved 970 at 8 h).

At the extreme other end, patient 3 (Figure 6) had nearly the maximum CSI he could achieve in 8 h (970). There is no room to improve his sleep in order to decrease his required time in bed and treatment needs to focus on managing excessive sleepiness.

Following the same logic, it may be reasoned that excessive wake time in an 8-h study in a patient whose CSI during the study is average or high, may be due to the patient achieving his normal sleep need in a shorter than the recommended time in bed and spends the rest of bedtime awake. On the other hand, a low CSI under the same circumstances in a non-sleepy patient suggests low sleep pressure throughout, which may be a hyperarousal state (see ORP type 1,3, below).

### Opportunities for research

- The above interpretations and management suggestions assume that an increase of one unit of CSI has the same restorative function regardless of whether it is generated by more sleep time or more sleep depth. This is an assumption that needs to be proven. The easy availability of the quantitative CSI makes it possible to address this fundamental question as well as other aspects of the restorative function of sleep.
- CSI provides an opportunity for determining personalized sleep need. Thus, measuring CSI in a subject who feels refreshed during a period of ad lib sleep on a sustained basis (e.g., vacation) would determine his/her total amount of sleep need. This would then represent the subject's sleep target needed under other conditions where he/she has non-restorative sleep, with the target reached through extension of regular time in bed or improvement in sleep depth via appropriate therapy, as the case may be.

D) ORP-related Sleep Architecture (The ORP Histogram): This is the most informative way of presenting ORP results (see Histograms in Figure 4). Rather than reporting % of time in different conventional sleep stages, percent of time in different ORP deciles is displayed as a histogram (7). The striking contrast between the three histograms in Figure 4, despite normal conventional architecture, illustrates the sensitivity of this approach. Such plots provide easy to recognize patterns that can be categorized, with the categories studied to determine their association with clinical outcomes. The patterns also allow caregivers to formulate hypotheses regarding the likely underlying mechanisms of the patient's complaints, which can be pursued by history or appropriate tests.

Based on susceptibility to arousal (Figures 2A, B) (1, 5) deciles 1 and 2 reflect the fraction of time spent in deep and very deep sleep (ORP < 0.5), while decile 10 (ORP >2.5) reflects time in full wakefulness (Figure 1). In between these two extremes the different deciles represent (from left to right), decreasing levels of sleep depth (deciles 3 and 4; ORP 0.50–1.00, Figures 1, 2) and transitional sleep with features of both sleep and wakefulness (deciles 5–7, ORP 1.00–1.75). Epochs in deciles 8 and 9 (ORP 1.75–2.25) are usually scored wake but they contain some sleep features (e.g., theta waves or periods of micro-sleep that are <15 s; Figure 1A) and reflect drowsy wake states (7).

**Figure 7:**
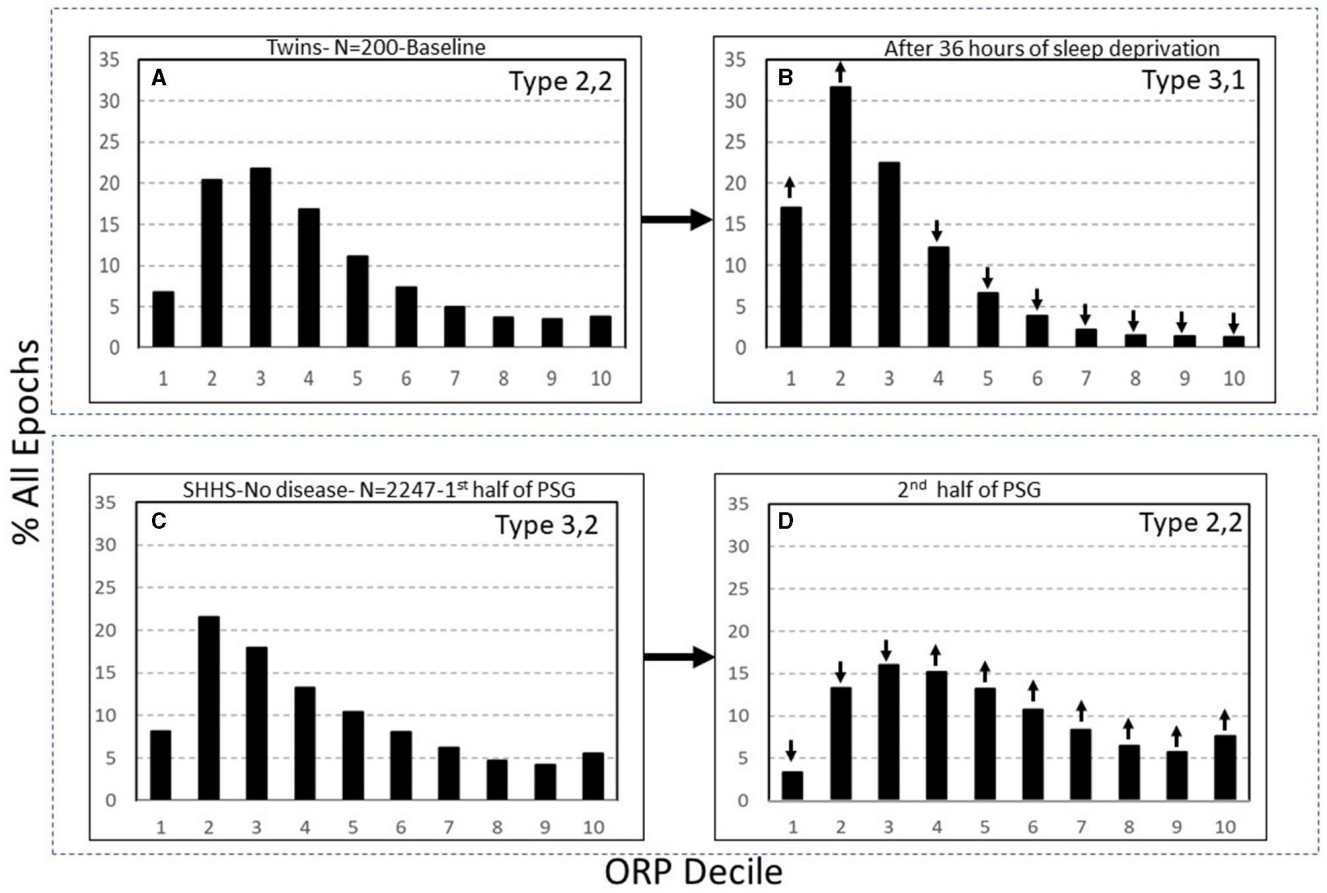
**(A, B)** Odds ratio product (ORP) architecture in 200 healthy participants in before and following 36 h of sleep deprivation. Note the remarkable leftward shift in the ORP distribution. **(C, D)** Comparison of ORP-architecture in the first and second halves of the night in Sleep Heart Health Study (SHHS) subjects with “No OSA or Insomnia.” An opposite shift is evident. ↓ and ↑, significant increase or decrease relative the same decile in the reference panel (*p* < 1E−10 in all). PSG, polysomnogram (From reference: Younes M, Gerardy B, Pack AI, Kuna ST, Castro-Diehl C, Redline S, et al. Sleep architecture based on sleep depth and propensity: patterns in different demographics and sleep disorders and association with health outcomes. *Sleep*. (2022) 45:59.).

Categorization of these patterns is based on the relation between times spent in deep sleep and in full wakefulness in response to pure changes in sleep pressure (7). In response to a pure increase in sleep pressure, as in after sleep deprivation, the histogram shifts to the left, with deep sleep increasing and full wakefulness decreasing, while in response to decreased sleep pressure, as in later in the sleep period, the opposite happens (Figure 7) (7). This paradoxical relation between deep sleep and full wakefulness is put to use as follows:

The full ranges of % deep sleep (deciles 1 + 2) and % full wakefulness (decile 10) were determined in 3,585 subjects of the Sleep Heart Heath Study (SHHS) who had >7 h of total recording time (7). The mid-range (25^th^-75^th^ percentile) for each variable (deep sleep and full wakefulness) was determined. Values in the lowest quartile of each range were assigned a rank of 1, values in the mid-range were assigned a rank of 2, and values in the highest quartile were assigned a rank of 3. A two-digit number was assigned to each PSG based on these two digits. Thus, type 1,3 describes a PSG with % deep sleep in the lowest quartile and % full wakefulness in the highest quartile, and so on. Nine types were, accordingly, categorized (1,1, 1,2, …3,3). Based on response to pure changes in sleep pressure (Figure 7), types with paradoxical relation between deep sleep and full wakefulness (e.g., 1,3 or 3,1) are consistent with low (type 1,3) or high (type 3,1) sleep pressure, respectively. When the two variables are both in the high or low quartile (e.g., 1,1 or 3,3), the type is not consistent with pure changes in sleep pressure.

In summary, assigning a two-digit number to ORP distribution makes it possible to easily appreciate the underlying pathophysiology. Thus, low first digit and high third digit (i.e., 1,3) signify a disorder associated with low sleep pressure across the night, and vice versa for type 3.1. When both digits are low (i.e., 1,1) the decreased amount of deep sleep is not due to low sleep pressure since there was little time in full wakefulness, and suggests a sleep disrupting disorder. And when both digits are high (i.e., 3,3) the excessive amount of full wakefulness is not due to low sleep pressure across the night (e.g., hyperarousal) since there was plenty of deep sleep. The other advantage is that these 9 patterns do not overlap (i.e., are mutually exclusive) which, unlike differences in times of conventional stages, limits the possible underlying mechanisms of sleep complaints.

**Figure 8:**
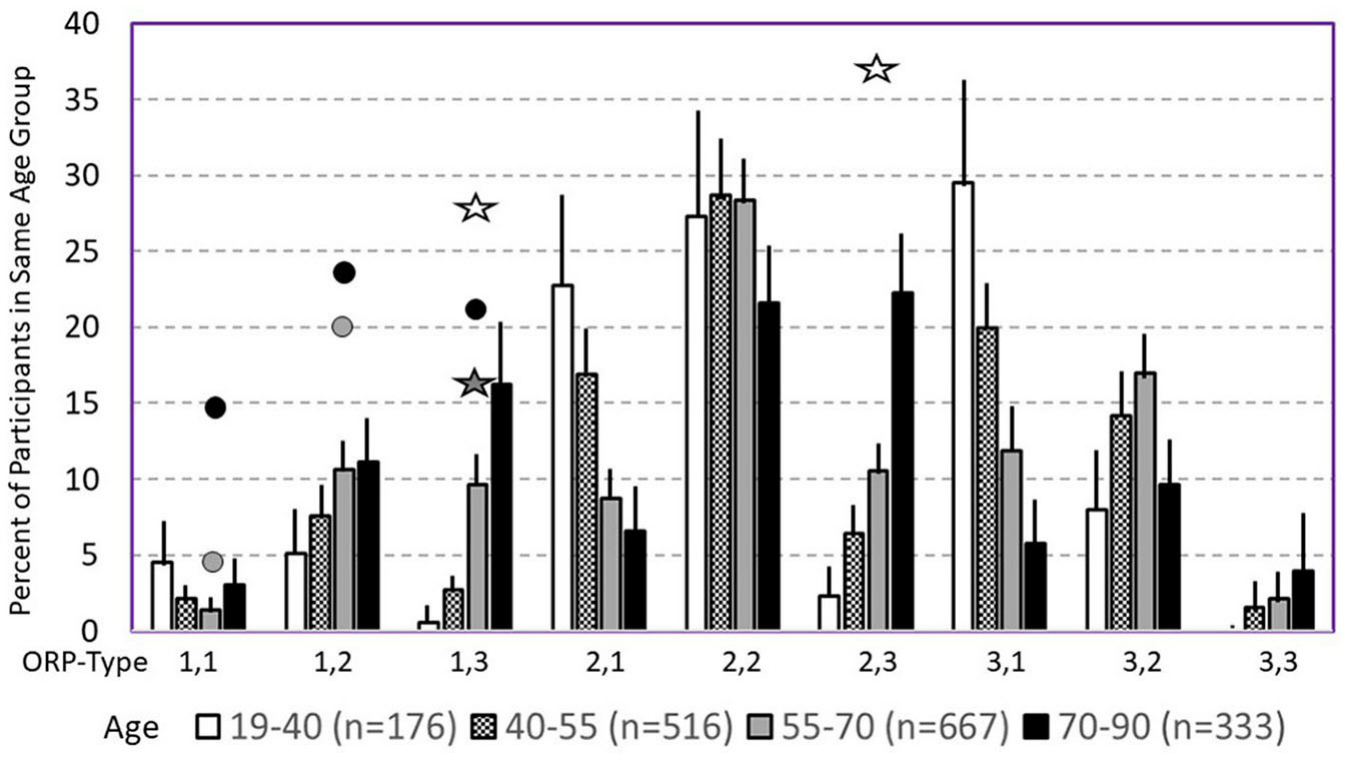
Prevalence of different odds ratio product (ORP) types in different age groups in the Sleep Heart Health Study. Lines are upper margin of error (95% confidence interval). Solid circles, values found in participants with severe (gray circle), and very severe OSA (black circles) in the different ORP types. White stars, values found in participants with insomnia and short sleep duration. Dark stars, values found in participants with insomnia plus OSA. All symbols are plotted against the 55–70 age group (gray columns) since average age in all clinical groups fell in this range. Where no symbols are shown above a given ORP type, the prevalence of the type is within the confidence interval of participants with no OSA or insomnia (From reference: Younes M, Gerardy B, Pack AI, Kuna ST, Castro-Diehl C, Redline S, et al. Sleep architecture based on sleep depth and propensity: patterns in different demographics and sleep disorders and association with health outcomes. *Sleep*. (2022) 45:59.).

The following section describes the clinical associations and likely mechanisms of the different ORP types (7), beginning with the most clinically relevant types. Table 2 gives an overview of the distribution of the different types in different clinical phenotypes in the SHHS, and Figure 8 shows the frequency of different types in different age groups in the same study (7).

**Table 2:** Distribution of different ORP architecture types in clinical categories.

**ORP type**	**Sleep heart health study**								**All**
	**“No disease”**	**Obstructive sleep apnea**				**Insomnia**			
		**Mild**	**Mod.^a^**	**Sev.^b^**	**V. sev.^d^**	**NSD^c^**	**SSD^e^**	**+OSA^c^**	
1,1	30 (2.0)	30 (3.1)	13 (3.5)	7^a^ (5.0)	11^d^ (15.1)	6 (3.3)	0 (0)	10 (4)	107
1,2	147 (9.7)	118 (12.1)	53 (14.2)	29^b^ (20.9)	18^b^ (24.7)	26 (14.4)	5 (6.3)	29 (11.6)	425
1,3	131 (8.6)	88 (9.0)	40 (10.7)	15 (10.8)	16^b^ (21.9)	9 (5.0)	23^d^ (28.8)	43^b^ (17.3)	365
2,1	168 (11.1)	106 (10.9)	37 (9.9)	16 (11.5)	3 (4.1)	21 (11.7)	3 (3.8)	23 (9.2)	377
2,2	406 (26.8)	266 (27.3)	102 (27.3)	40 (28.8)	13 (17.8)	64 (35.6)	9 (11.3)	57 (22.9)	957
2,3	177 (11.7)	128 (13.2)	54 (14.4)	16 (11.5)	4 (5.5)	10 (5.6)	31^d^ (38.8)	38 (15.3)	458
3,1	201 (13.2)	104 (10.7)	39 (10.4)	11 (7.9)	5 (6.8)	20 (11.1)	1 (1.3)	21 (8.4)	402
3,2	219 (14.4)	114 (11.7)	36 (9.6)	5 (3.6)	1 (1.4)	23 (12.8)	3 (3.8)	20 (8.0)	421
3,3	35 (2.3)	19 (2.0)	3 (0.8)	0 (0)	2 (2.7)	1 (0.6)	5 (6.3)	8 (3.2)	73
Total	1,517	973	374	139	73	180	80	249	3,585

OSA, obstructive sleep apnea; NSD, normal sleep duration; SSD, short sleep duration; Mod., Sev., V.Sev., are moderate, severe, and very severe OSA, respectively. values are number of subjects with the type indicated; numbers in brackets indicate the percent of subjects in each clinical category with the type indicated. Differences between values in each category and the “No-disease” category were evaluated by the Chi-square test and their significance is indicated by superscripts in the column heading.

a *p* < 0.02;

b *p* < 0.0001;

c *p* < 1.E-5;

d *p* < 1.E-10;

e *p* < 1.E-25. From Table 3 in reference Younes et al. (7).

Type 1,1 (Figure 4B, right): The low amount of deep sleep suggests either low sleep pressure or a disorder that interrupts progression to deep sleep. However, the low amount of full wakefulness does not support the existence of low sleep pressure (7). Accordingly, the most likely mechanism of this pattern is a disorder that interferes with progression to deep sleep and, likely as a consequence, may be associated with high sleep pressure (e.g., excessive sleepiness) (7). The following findings support this conclusion:

- This pattern is rare (2%) in community dwellers free of OSA or insomnia (7).
- Its frequency increases exponentially as OSA severity increases (3.1%, 3.5%, 5%, and 15.1%, respectively, with mild (AHI 5–15 h^−1^), moderate (AHI 15–30 h^−1^), severe (30–50 h^−1^), and very severe (AHI >50 h^−1^) OSA) (Table 2) (7).
- Its frequency is also very high in critically ill, intubated un-sedated patients in the intensive care unit (33%) (17), where OSA is not a factor but other factors that preclude progression to deep sleep are in abundance (30).
- It is one of only three (of nine) ORP types in which CPAP improves sleep among patients with OSA (6).
- Among patients with OSA it is the ORP pattern associated with highest average ESS (11.3±5.4), highest frequency of ESS>10 (67.9%), and highest frequency of ESS >17 (15.5%) (6).
- Among all ORP types it is associated with the lowest mental [SF36(M)] and second lowest physical [SF36(P)] quality of life scores (7).

These observations support the notion that this pattern results from a disorder that interferes with progression to deep sleep that is frequently associated with excessive sleepiness, with OSA being the most recognized, but not the only, example of such a disorder. Thus, finding pattern 1,1 in a patient with sleep symptoms and no obvious sleep pathology that can account for it in the PSG (e.g., subject in Figure 4B) suggests the presence of other sources of frequent arousal stimuli (skin, musculoskeletal, gastrointestinal, …etc.).

Type 1,2: This type is similar to type 1,1 except that full wakefulness accounts for up to 12% of TRT instead of being <3.4% in type 1,1 (7). As in pattern 1,1, its frequency increases with OSA severity (Table 2) (6, 7), and it is one of the three types where sleep improves on CPAP (6). It is also associated with higher ESS and lower quality of life (7). Accordingly, it is considered to have the same underlying mechanism as type 1,1. It is, however, more common in the community, occurring in 9.7% of subjects with no OSA or insomnia, as opposed to 2% for type 1,1 (Table 2) (7). Thus, it is more likely to be encountered in patients with no OSA. It is still not clear, however, if it is associated with excessive sleepiness and poor quality of life if not associated with OSA.

### Opportunities for research

- The long term impact of OSA on health outcomes is currently uncertain (60). It is likely that negative health outcomes occur in only a minority of patients such that they are obscured when diluted with a large number of patients who are not so affected. As a corollary, treatment of OSA may benefit only a subset of patients and this benefit is obscured when outcomes of therapy studies are performed on unselected patients. Currently, efforts are directed at identifying patients whose long term health outcomes are adversely affected and, by extension, who might benefit from CPAP or other OSA therapy. Conventional sleep study metrics, including AHI, are not very helpful in this regard (60). Recently, other markers such as hypoxic burden and presence of excessive somnolence have been associated with increased risk of cardiovascular events but the results have been inconsistent in different cohorts (29, 31–35). The discovery that only two ORP types, 1,1 and 1,2, are associated with poor sleep quality and associated excessive sleepiness, provides an opportunity to determine whether these two types selectively benefit from OSA therapy.
- Similarly, given the myriad causes of excessive sleepiness, at least as measured by the Epworth Sleepiness Scale (ESS), presence of somnolence in a patient with OSA does not indicate that somnolence is caused by OSA (6). In fact, average ESS does not begin to increase until AHI is >45 h^−1^ and even then the increase is minimal (6). Determining if sleepiness in sleepy patients with OSA improves preferentially in those with ORP types 1,1 and 1,2 would be worthwhile.
- Given that the selective improvement in sleep on CPAP in types 1,1 and 1,2 was found in split sleep studies, additional prospective studies while patients are on long term CPAP are needed to confirm these findings.

**Figure 9:**
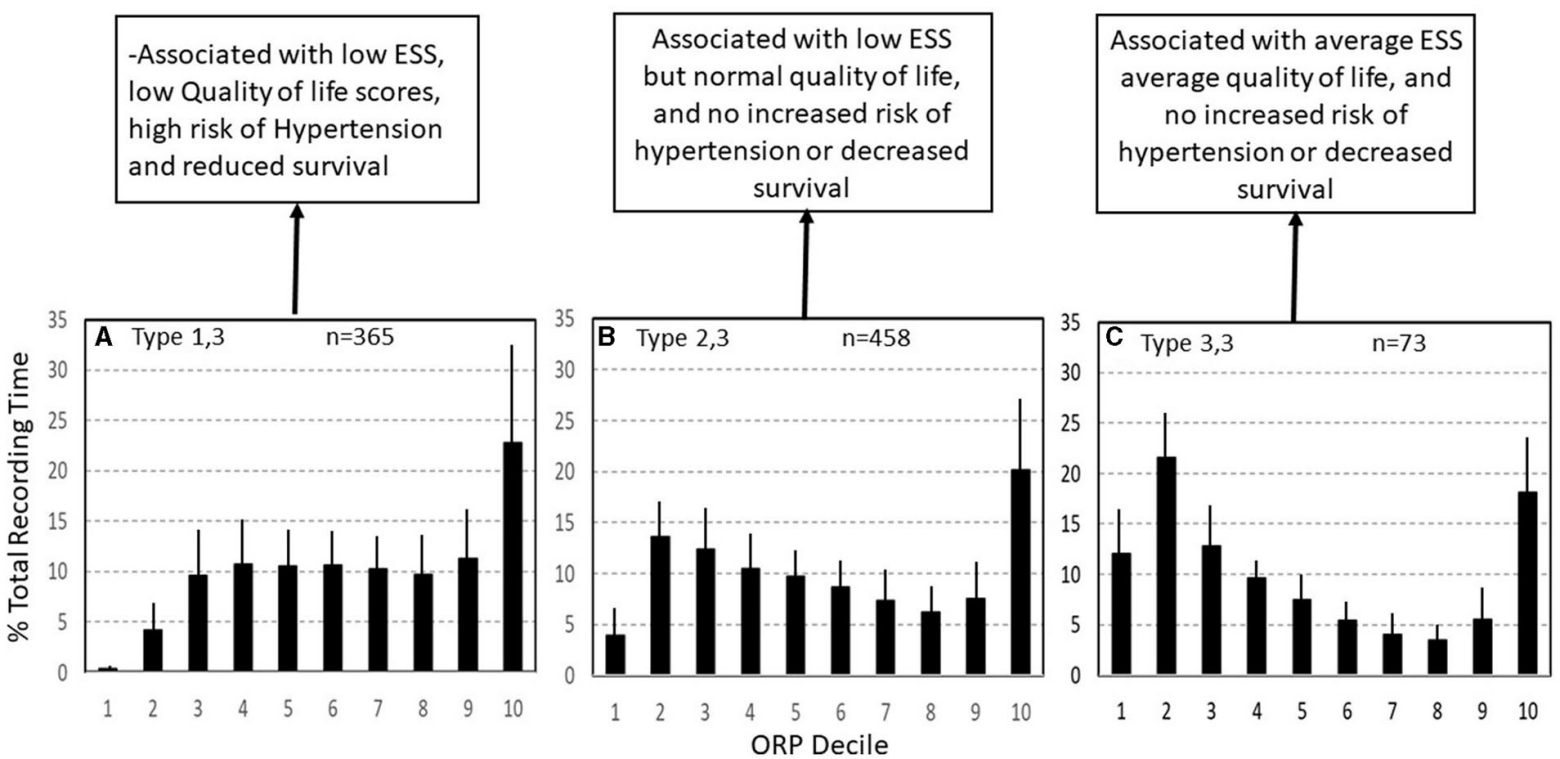
Three ORP types found in subjects with excessive time in full wakefulness (decile 10). In type 1,3 **(A)** there is little deep sleep. In type 2,3 **(B)** time in deep sleep is normal, and in type 3,3 **(C)** time in deep sleep is above average. Types 1,3 and 2,3 are the most common types in insomnia with short sleep duration. ESS, Epworth Sleepiness Scale (From reference: Younes M, Gerardy B, Pack AI, Kuna ST, Castro-Diehl C, Redline S, et al. Sleep architecture based on sleep depth and propensity: patterns in different demographics and sleep disorders and association with health outcomes. *Sleep*. (2022) 45:59.).

Type 1,3 (Figure 9A): This pattern is most relevant to patients with excessive wake time (low sleep efficiency). The paradoxical relation between deep sleep (very little) and full wakefulness (excessive) in this type is consistent with low sleep pressure *across the night*. Given the multiple mechanisms of low sleep pressure, this pattern is ubiquitous, occurring in asymptomatic subjects and in association with various sleep disorders (Table 2) (7). In a large community cohort (Sleep Heart Health Study; SHHS) pattern 1,3 was present in 365 of 3,585 (10.2%) of all subjects (Table 2). Of these, 131 (35.8%) occurred in subjects with no insomnia or OSA (7). Of the remaining 234 subjects with this pattern 202 subjects (87%) had concomitant OSA (Table 2) (7). Of these, only 43 (21%) also had concomitant insomnia (Table 2) (7), thereby meeting the criteria of COMISA (Comorbid insomnia and sleep apnea) (36).

It should be pointed out that the frequency of type 1,3 in patients with mild, moderate, and severe OSA is not significantly different than that of subjects with no OSA and that even in very severe OSA (AHI >50 h-1), the number of patients with this type (16, Table 2) exceeded that expected from values in no OSA (6, Table 2) by only relatively few patients (10), several of whom had insomnia symptoms but in whom symptoms were less frequent than 3 times per week. Thus, the extra patients with type 1,3 (*n* = 10) may have been examples of COMISA. Furthermore, in a separate study on patients with insomnia and excessive wake time, there was no difference in ORP in any sleep stage between those with and without mild-moderate OSA (8), and in a separate study, wake time remained high, albeit somewhat lower, in patients with type 1,3 and insomnia when treated with CPAP (6). Accordingly, when type 1,3 and OSA coexist, the excessive wake time likely reflects a state of low sleep pressure, independent of OSA.

Consistent with earlier findings that wake time increases with age (37, 38), type 1,3 increases dramatically in frequency with age in asymptomatic subjects, from <2% (95% percentile) in those <40 years to 20% in those over 70 years (Figure 8) (7). The increase in wake time with age is primarily in the ORP range of full wakefulness (decile 10), with much smaller increases in drowsy wakefulness (deciles 8 and 9) (7), suggesting that the excess wake time in older people is due to age-related decrease in sleep pressure (or need) rather than to age-related diseases that impair sleep (7). It follows that finding this pattern need be of concern only if associated with insomnia or if the patient is young even in the absence of insomnia, where it may indicate a disorder of low sleep pressure, for example a latent hyperarousal state or excessive napping.

In summary, the above findings suggest that OSA is not causally related to excessive wake time when the two conditions coexist except possibly in very severe OSA. Even when the associated OSA is severe, it is possible that the low sleep pressure in this pattern may be contributing to OSA severity, rather than the other way around (8). By correcting upper airway instability CPAP use in such combined cases is associated with improved sleep depth but is not expected to normalize wake time (6).

The relation between type 1,3 and insomnia is complex. Of 365 subjects with this pattern in the SHHS, only 75 (20.5%) met the accepted definition of insomnia (Table 2) despite the excessive wake time (Table 1) (7). On the other hand, the frequency of type 1,3 in subjects with COMISA (17.3%) and in those with insomnia and short sleep duration (Insomnia SSD; 28.8%, Table 2), was significantly higher than in those without insomnia (8.7%) or in those with paradoxical insomnia (Insomnia with normal sleep duration (NSD); 5%, Table 2), suggesting a causal relation between the excessive wake time in this type and the patient's symptoms.

For the entire SHHS cohort type 1,3 was associated with the lowest SF36 (P) and the third lowest SF36 (M) (7). These associations persisted after adjusting for age, gender, body mass index (BMI), AHI, and insomnia. In addition, type 1,3 was associated with significantly lower SF36 (P) and SF36 (M) in subjects with no OSA or insomnia after adjusting for age, gender, and BMI (7). Thus, this type is the most consequential with respect to quality of life, whether or not it is associated with OSA or insomnia. Type 1,3 was also associated with higher risk of hypertension and all-cause mortality in the same study (unpublished observations).

It is worth noting that despite the progressive increase in frequency of types 1,1, 1,2, and 1,3 with OSA severity (Table 2), other types not associated with poor sleep, sleep improvement on CPAP, or reduced quality of life (Types 2,1 to 3,3, see below) are also seen in OSA (Table 1). These types accounted for 75.8%, 71.6%, 63.3%, and 38.3% of all patients in mild, moderate, severe, and very severe OSA, respectively (Table 2) (7).

### Opportunities for research

1) Further studies are needed to determine if long term CPAP use improves clinical outcome in patients with mild-moderate OSA associated with excessive wake time since the impairment in sleep depth at this level of severity is minimal (6).

2) Sleep in Critically Ill Patients: It is well-known that sleep is very poor in critically ill patients in intensive care units (39–42). Independent of critical illness, poor sleep adversely affects several organ functions that are critical for recovery and liberation from mechanical ventilation (immune function (43, 44), respiratory control (45), neuroendocrine and metabolic function (46–48), cardiovascular responses (49), mental health (50, 51). It is therefore likely that poor sleep contributes to poor outcome in such patients (30, 52). An important question, therefore, is whether normalization of sleep improves clinical outcomes in these patients. Critically ill patients are routinely administered different kinds of sedatives to help them sleep. However, whether these sedatives result in normal sleep or simply act as CNS depressants is not known.

ORP was recently used to study sleep in critically ill patients (15–17). In one study, those with little or no time in full wakefulness during 15 h of monitoring were less likely to pass a weaning trial (15). In another study on un-sedated stable patients prior to extubation, ORP types 1,1, 1,2, and 1,3 were present in 68% of patients, by contrast to a frequency of 25.8% in the general community (17). Of these three types, type 1,1 was the most frequent (33.0%) followed by type 1,3 (22.0%) (17). Furthermore, these abnormal ORP patterns were found with similarly high frequency (77%) among a separate cohort of intensive care unit (ICU) survivors, with little improvement 6 months after discharge (17). These findings strongly suggest that poor sleep in critically ill patients, prior to attempted extubation, is largely due either to arousal stimuli that preclude progression to deep sleep (types 1,1 and 1,2) or to a hyperarousal state (type 1,3), and that these abnormalities persist even months after discharge (17).

In a third study, the impact of *mild* sedation with propofol and dexmedetomidine on ORP architecture was studied (16). These two agents caused a leftward shift in the ORP distribution toward the normal pattern in all patients, with pattern becoming normal or almost normal in most patients. On average, ORP architecture approached normal distribution. Importantly, apart from normalizing ORP distribution, the spectral pattern of the EEG at any given ORP was indistinguishable from that in natural sleep outside the ICU, suggesting that these agents, in appropriate doses, are capable of producing normal sleep rather than CNS depression (16).

Collectively, these three studies indicate that poor sleep in the ICU is mostly related to abnormal arousal stimuli, or a hyperarousal state, and that sleep can be normalized by the appropriate kind and amount of sedation. Also given that ORP can be measured and displayed in real time (53) it would be feasible to control the sedative dosage using ORP feedback. It would be of great interest to determine whether, using such feedback, clinical outcomes improve by implementing a sustained period of sleep (e.g., corresponding to normal total sleep time) with some variation in sleep depth to simulate the different sleep cycles seen normally, and adjusted to coincide with nighttime to maintain a normal circadian rhythm.

Type 2,3 (Figure 9B): As in type 1,3, type 2,3 is characterized by excessive time in full wakefulness (Figure 9B) and a high frequency in insomnia SSD (38.8%) but not insomnia NSD (Table 2). Therefore, the excessive time in full wakefulness likely contributes to insomnia symptoms. Type 2,3 is the most frequent type in insomnia SSD (Table 2). However, in marked contrast to type 1,3, type 2,3 is associated with normal amount of deep sleep (sum of deciles 1 and 2; 17.6 ± 4.9% vs. 4.6 ± 3.2%, *p* < 0.0001; Figure 9B). It is also not associated with reduced quality of life, (7) or hypertension or mortality (unpublished). These findings, along with a normal (average) ESS (6, 7) and no associated poor health outcome suggest that this type occurs in people who obtain enough restorative sleep but stay in bed longer than they need to Younes et al. (7).

**Figure 10:**
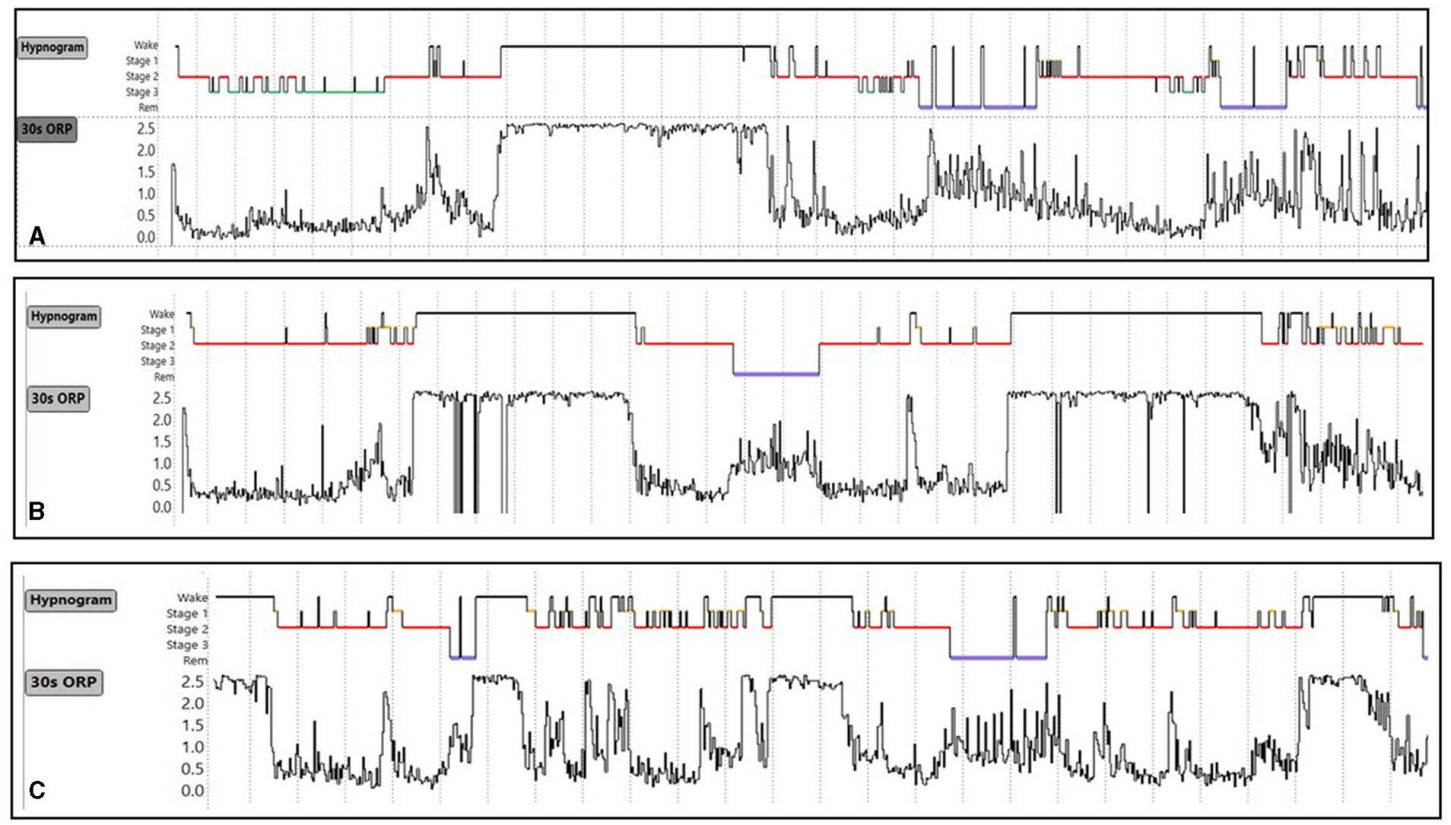
**(A–C)** Conventional sleep hypnograms and 30-s odds ratio product (ORP) results in three subjects with ORP type 3,3 [high amounts of deep sleep (ORP < 0.5) as well as high amounts of full wakefulness (ORP >2.25)] showing the different locations of excessive time in full wakefulness within the sleep study. Unpublished data.

Type 3,3 (Figure 9C): As its 2-digit number indicates, this type is associated with high amounts of deep sleep as well as full wakefulness (Figure 9C). Its frequency was very low in all age groups in subjects with no OSA or insomnia in the SHHS (Figure 8) and its frequency did not increase with OSA severity and/or insomnia (Table 2). ESS and quality of life are average (7). The location of the fully wake time within the sleep study is highly variable and may consist of one long period early, late or in mid-region, or multiple shorter periods within sleep period time (Figure 10). This type suggests a circadian disorder or an individual who meets his sleep need in less time than time in bed (short sleeper). Multiple short awakenings may also be related to urination. Wake periods often start suddenly from deep or REM sleep (Figure 10), which may suggest a parasomnia. Enquiry about these possible causes would be appropriate in patients with this type.

### Opportunities for research

#### Research in insomnia

At present, sleep studies are not recommended for patients with insomnia as they are felt to contribute little to clinical management and may exclude many patients with primary insomnia (54). The use of ORP in patients with insomnia has, however, identified several phenotypes that differ in health outcomes and likely underlying mechanisms and, potentially in response to therapy. These findings advocate for use of PSG in patients with insomnia, if only for research purposes.

Insomnia with Normal Sleep Duration: As expected we found no difference in conventional indices between insomnia with normal sleep duration and subjects with no insomnia (7). Also, apart from one interesting finding, there were no differences in ORP architecture between these patients and those with no insomnia (Table 2) (7). However, in marked contrast to insomnia with short sleep duration, time in full wakefulness (decile 10) was significantly lower than in patients with no insomnia (7). To the extent that less time in full wakefulness is suggestive of higher sleep pressure (Figure 7), this observation suggests that, notwithstanding the lack of difference in distribution of ORP types (Table 2), sleep may have been of lower quality in these patients. Further investigation into differences in sleep microstructure is warranted.

Insomnia with Short Sleep Duration: Vgontzas et al. reported that insomnia with SSD is the most biologically severe form of insomnia, being associated with a high risk of hypertension, diabetes, cognitive impairment, and mortality (55–59). The current findings indicate that ORP architecture in insomnia SSD includes several distinct phenotypes that share excessive wake time but differ in other respects: (a) ORP type 1,3 with poor sleep along with poor health outcomes (Figure 9A); (b) ORP type 2,3 with normal sleep quality and no adverse health outcomes (Figure 9B); (c) Type 3,3 with better than average sleep quality and no adverse health outcomes (Figure 9C); (d) Other uncommon types in which excessive wake time is preferentially in the drowsy wake state (deciles 8 and 9) (Table 2). Of these, except for type 1,2, which accounts for 6.3% of these patients and is associated with slight reduction in SF36 (M), these types are also not associated with adverse health outcomes.

Thus, it is possible that the adverse effects described by Vgontzas et al. (55–59) stem from the increased representation of type 1,3 in this insomnia category (Table 2). Also, given the different likely mechanisms of these various phenotypes (7), it is possible that response to insomnia therapy may differ among these phenotypes. It would be of considerable clinical importance to confirm these findings and to determine if these types respond differently to insomnia treatment modalities.

*Types 3,1 and 2,1:* Type 3,1 is the prototypical pattern of *uncomplicated* high sleep pressure (Figures 2C, 7B). Except for a lesser amount of deep sleep (19.8 ± 5.2% vs. 41.1 ± 9.7%TRT) type 2,1 shares all characteristics and associations as type 3,1. Thus, their frequency is highest in young adults (>24% of adults <40 years; Figure 8) and decreases progressively with age to <7% in subjects >70 years (Figure 8). On average, in the SHHS, they occurred with similar frequency in adults with no OSA or insomnia (13.2 and 11.1%, respectively; Table 2) and, reflecting the impact of severe OSA in preventing progression to deep sleep, frequency decreased as OSA severity increased, becoming uncommon (< 7%) in very severe OSA (Table 2). Also as expected, their frequency is much reduced in insomnia SSD but not in insomnia NSD (Table 2). When associated with OSA, their presence indicates that sleep depth is not degraded by the disorder. This is supported by the fact that sleep depth does not improve, or deteriorates, when CPAP is used (acutely) in patients with OSA and this ORP type (6).

Reflecting the fact that most subjects with these two types have no sleep disorder or have OSA that does not interfere with progression to deep sleep (see above and Table 1), ESS is not significantly increased (6, 7). However, given their similarity with the pattern seen with sleep deprivation (Figure 7B), and their high frequency in patients with idiopathic hypersomnia (Patient 3, Figure 6), (27) occurrence of type 3,1 in subjects with excessive sleepiness (e.g., Figure 4C), and particularly in older individuals, would suggest that the patient may not be getting sufficient sleep because of poor lifestyle or excessive sleep need (idiopathic hypersomnia; see Cummulative Sleep Index, CSI; above), and such disorders need to be excluded before discounting insufficient sleep as the reason for excessive sleepiness.

Type 2,2 (Figures 2A, 6A, D): This is the most common type among subjects (in the SHHS) with no OSA or insomnia (26.8%, Table 1), and its frequency is nearly the same at all OSA severity levels and both insomnia types (Table 2). ESS and quality of life indices are average (7). The average amounts of deep sleep and full wakefulness argue against high or low sleep pressure. Accordingly, this type almost certainly reflects normal sleep. If associated with symptoms suggestive of a sleep disorder (sleepiness, non-restorative sleep, insomnia) the symptoms are not likely due to a sleep abnormality (7).

Type 3,2 (Figure 7C): This type differs from type 2,2 in having more deep sleep while the amount of full wakefulness is average, making it less likely that sleep pressure is high. It is most common in subjects with no OSA or insomnia (Table 2) and its frequency decreases as OSA severity increases and in insomnia SSD (Table 2). It is also not associated with increased sleepiness or poor quality of life (7), and when associated with OSA sleep depth is not responsive to CPAP (6). These findings suggest that this is a normal pattern.

In summary, there are four patterns that predominate in subjects with no obvious sleep pathology and their frequency either decreases or is unchanged in the presence of OSA or insomnia (Types 2,1, 3,1, 2,2, and 3,2). They are statistically not associated with excessive sleepiness or poor quality of life. Accordingly, such patterns likely represent normal sleep except when they are found under specific circumstances such as types 2,1 or 3,1 in an older subject or in a subject with objective excessive sleepiness. Type 2,3 also appears to be a normal pattern except for its frequent occurrence in insomnia with short sleep duration, where it may be a milder variant of type 1,3 or, pending investigations, simply reflect staying in bed longer than needed. On the other hand, types 1,1 and 1,2 always warrant investigation into possible sources of frequent arousals. Type 1,3 is also of concern, except in old asymptomatic subjects, as it may indicate a disorder of low sleep pressure (e.g., hyperarousal, excessive napping…etc.).

## Conclusion

ORP is a continuous metric of sleep depth and wake propensity. It makes it possible to distinguish different wake states in the transition from full wakefulness to light sleep and different levels of sleep depth within the same conventional sleep stage. It has been extensively validated. It can be reported in graphic as well as numeric ways. When reported as percent of recording time spent in different ORP deciles the distribution patterns are distinct from each other and suggest different underlying mechanisms for patient symptoms. Using these patterns, different phenotypes have been found in patients with OSA, insomnia and idiopathic hypersomnia. These provide the bases for future research that could pave the way for improved management of these disorders.

## Data availability statement

The original contributions presented in the study are included in the article/supplementary material, further inquiries can be directed to the corresponding author.

## Ethics statement

Ethical review and approval was not required for the study on human participants in accordance with the local legislation and institutional requirements. Written informed consent from the patients/ participants or patients/participants legal guardian/next of kin was not required to participate in this study in accordance with the national legislation and the institutional requirements.

## Author contributions

MY: Conceptualization, Writing—original draft, Writing—review and editing.
